# Surgical Fixation of Distal Humeral Nonunion in an Elderly Patient Three Years After Injury: A Case Report

**DOI:** 10.7759/cureus.81653

**Published:** 2025-04-03

**Authors:** Takahiro Yamazaki, Yusuke Matsuura, Seiji Ohtori

**Affiliations:** 1 Orthopaedic Surgery, Chiba University, Chiba, JPN

**Keywords:** bone graft, distal humeral fracture, dual plating, elderly patient, internal fixation, nonunion

## Abstract

Distal humeral fractures are relatively rare and are known to have a high nonunion rate compared to other humeral fracture sites. Treatment options for nonunion include open reduction and internal fixation (ORIF) and total elbow arthroplasty (TEA). While TEA has been increasingly reported as a treatment for distal humeral nonunion in recent years, it has limitations, including weight-bearing restrictions. This case report describes an 82-year-old female with a distal humeral nonunion that had been untreated for three years. Despite her advanced age and the prolonged time since injury, successful treatment was achieved with ORIF using dual plating technique and iliac bone grafting. Bone union was confirmed four months postoperatively, and the patient regained excellent elbow function with no pain or instability. This case suggests that ORIF can be a viable option even for elderly patients with long-standing distal humeral nonunion when appropriate surgical techniques are employed and articular surfaces are preserved.

## Introduction

Distal humeral fractures are relatively rare, accounting for approximately 1-2% of all fractures [1,2]. They are complex fractures involving constraints of bone and soft tissue. These fractures show a bimodal distribution, occurring frequently in children and adults over 60 years of age [3]. By 2030, the incidence of distal humeral fractures in the elderly population is predicted to increase up to threefold [4].

Distal humeral fractures often fail to achieve bone union with conservative treatment, leading to delayed union or nonunion [5,6]. In fact, conservative management has been reported to have a nonunion rate six times higher compared to surgical treatment [6]. When nonunion occurs, daily activities are significantly impaired due to pain and elbow instability.

Despite advances in surgical techniques, nonunion after distal humeral fractures occurs in 8-25% of cases [4,7]. This is a higher frequency compared to other humeral fracture sites (less than 4% for proximal humerus and less than 2% for humeral shaft) [4,8]. The main causes of nonunion include bone fragility, insufficient blood supply to bone fragments, and scarring of periarticular soft tissues, which often makes surgical treatment challenging [9,10].

Treatment options for nonunion primarily include open reduction and internal fixation (ORIF) and total elbow arthroplasty (TEA) [11]. Internal fixation is chosen when the articular surface is preserved and bone quality is sufficient, while TEA is indicated when there is significant destruction of the articular surface and severe bone defects in the articular region, particularly in elderly female patients [12,13].

Recently, there have been several reports of TEA for nonunion after distal humeral fractures [13,14]. However, the long-term outcomes of TEA are not yet well established, and it has the disadvantage of requiring weight-bearing restrictions [15,16]. On the other hand, there are also reports that bone fixation is possible using locking plates with simultaneous soft tissue release when bone morphology is preserved [17,18].

This report presents a case of successful ORIF for nonunion of a distal humeral fracture in an elderly patient three years after the initial injury.

## Case presentation

The patient was an 82-year-old female who had sustained a right distal humeral fracture from a fall three years prior. Although surgery had been recommended, she had declined treatment due to work commitments. After retirement, she was referred to our hospital with complaints of elbow pain and instability.

Her medical history included hypertension, diabetes mellitus, and compression fractures, but she was independent in activities of daily living and had no cognitive impairment.

**Figure 1:**
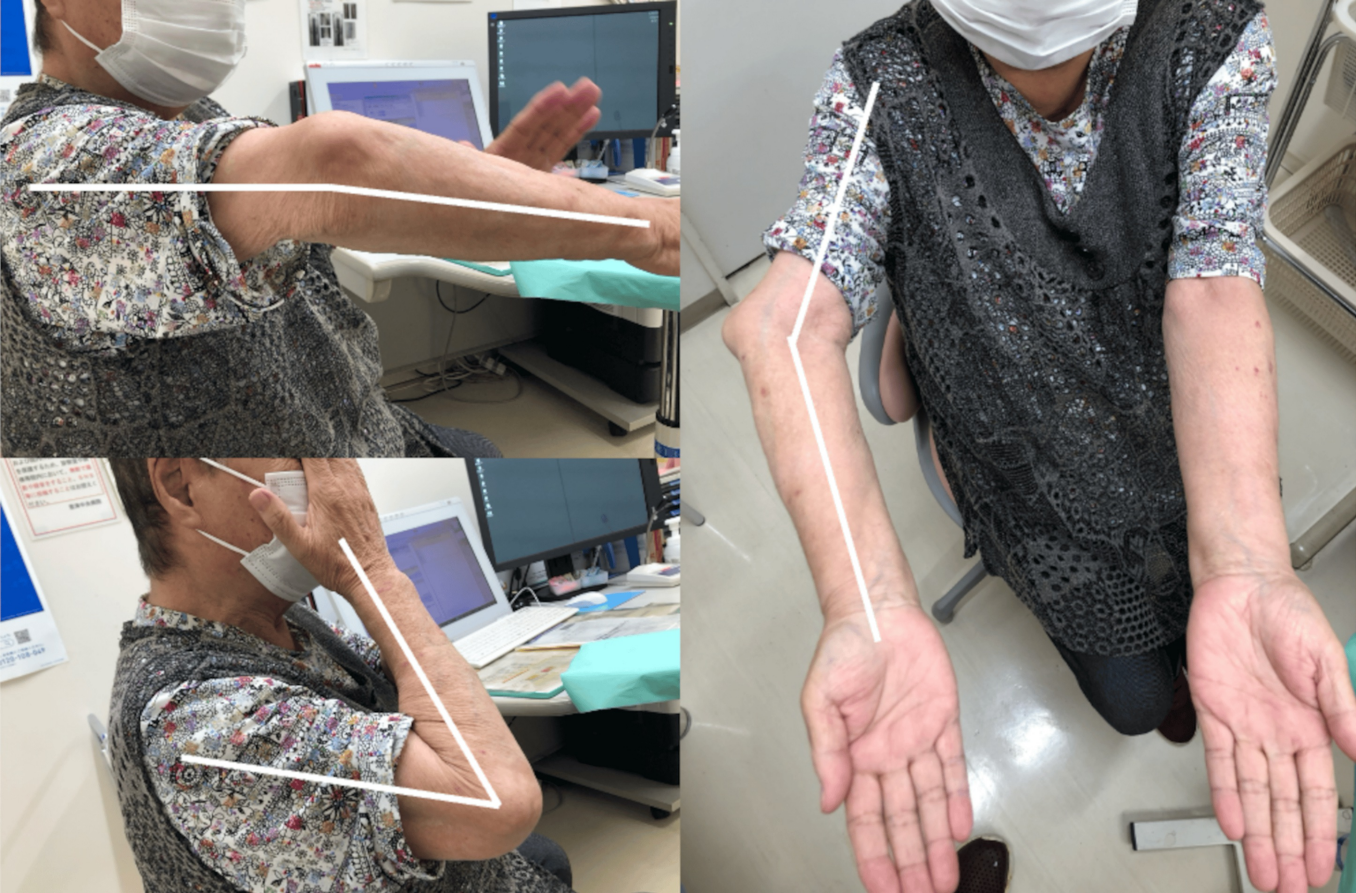
Preoperative Range of Motion and Appearance The range of motion was -5 degrees extension, 130 degrees flexion, with a carrying angle of -35 degrees, showing significant varus deformity.

She had no pain at rest but experienced pain during elbow movement and when performing a push-off motion to stand up, along with a sensation of elbow instability. The range of motion was -5 degrees extension, 130 degrees flexion, with a carrying angle of -35 degrees, showing significant varus deformity (Figure 1).

**Figure 2:**
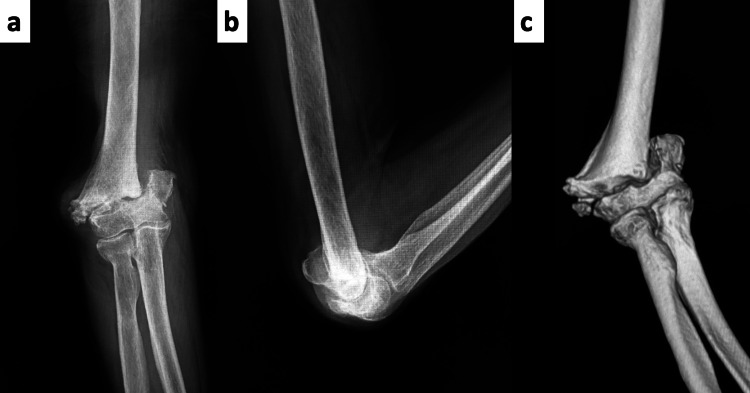
Preoperative Imaging (a) Anteroposterior (AP) view and (b) lateral view plain radiographs, along with (c) a preoperative 3D CT image, showed a nonunion of a transcondylar distal humeral fracture. While bone defects were significant, the articular surface remained intact.

Plain radiographs and CT images showed nonunion of a transcondylar distal humeral fracture. While bone defects were significant, the articular surface remained intact (Figure 2).

Since this was her dominant hand and she strongly desired surgical intervention, we decided to proceed with nonunion surgery. Though TEA was another surgical option, we chose ORIF first due to concerns about weight-bearing limitations with arthroplasty.

**Figure 3:**
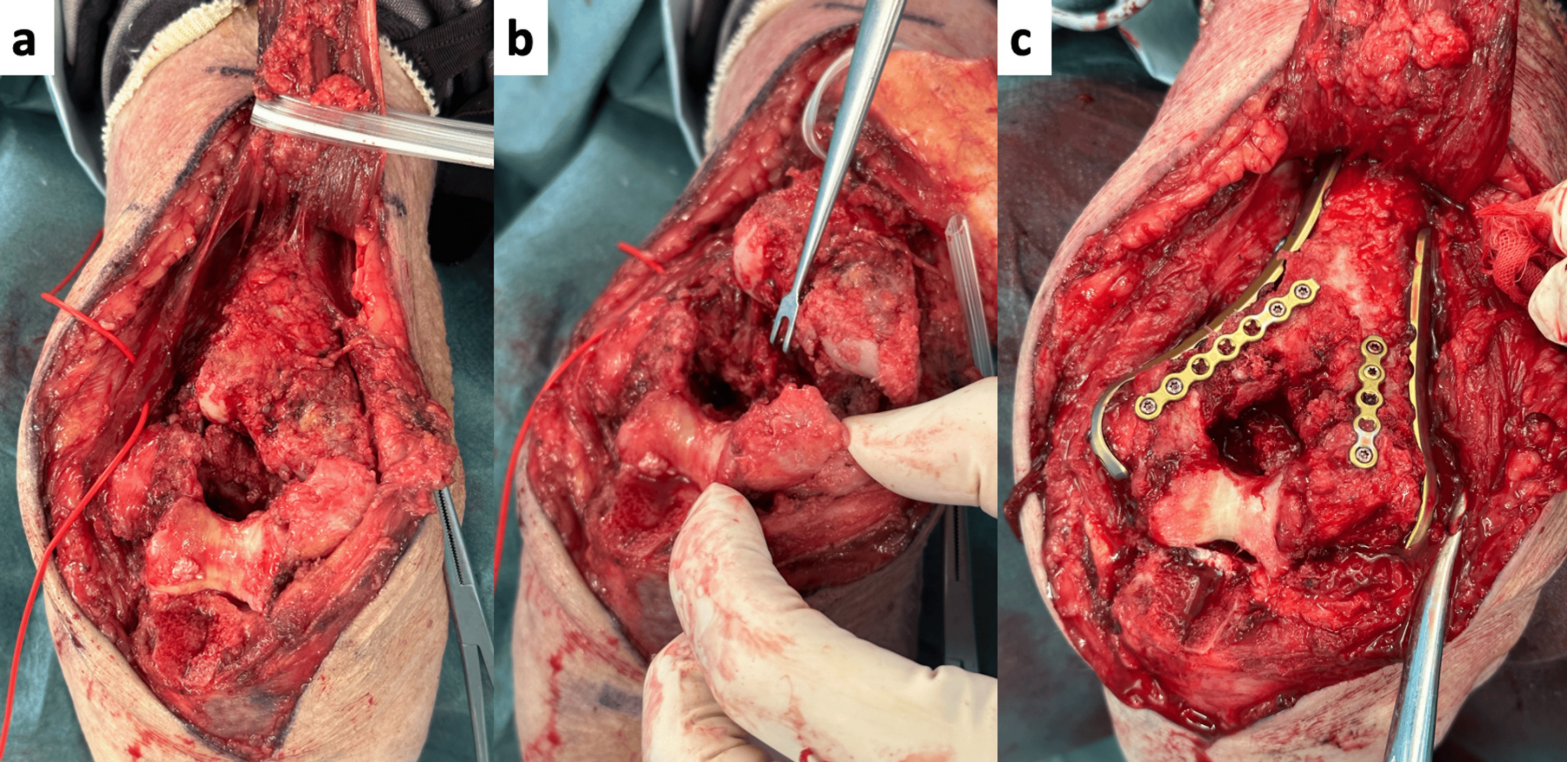
Intraoperative Findings (a) A Chevron osteotomy was performed for exposure. (b) The nonunion site was rounded and sclerotic. Decortication was performed as extensively as possible. (c) Bone grafts harvested from the iliac crest were placed in both the medial and lateral columns, and the grafts were secured with a mini-plate. Fixation was achieved with a Synthes Dynamic Humeral Plate (DePuy Synthes, a Johnson & Johnson Company, Raynham, MA, USA) in a 180° configuration.

**Figure 4:**
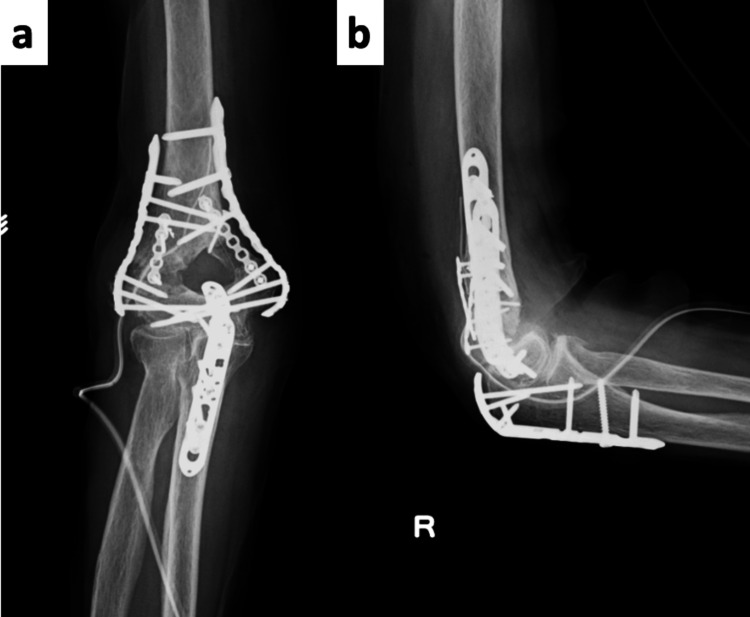
Postoperative Plain Radiographs (a) Anteroposterior (AP) view; (b) lateral view. Postoperative images demonstrate good reduction and stable fixation.

Surgery was performed under general anesthesia with the patient in a prone position. Using an olecranon osteotomy approach, we refreshed the nonunion site and performed iliac bone grafting. Double plating was applied from the medial and lateral sides, and additional reinforcement was provided with a mini-plate to secure the transplanted iliac bone (Figure 3). Rigid internal fixation with good alignment was achieved (Figure 4). A good range of motion was obtained intraoperatively without soft tissue release.

**Figure 5:**
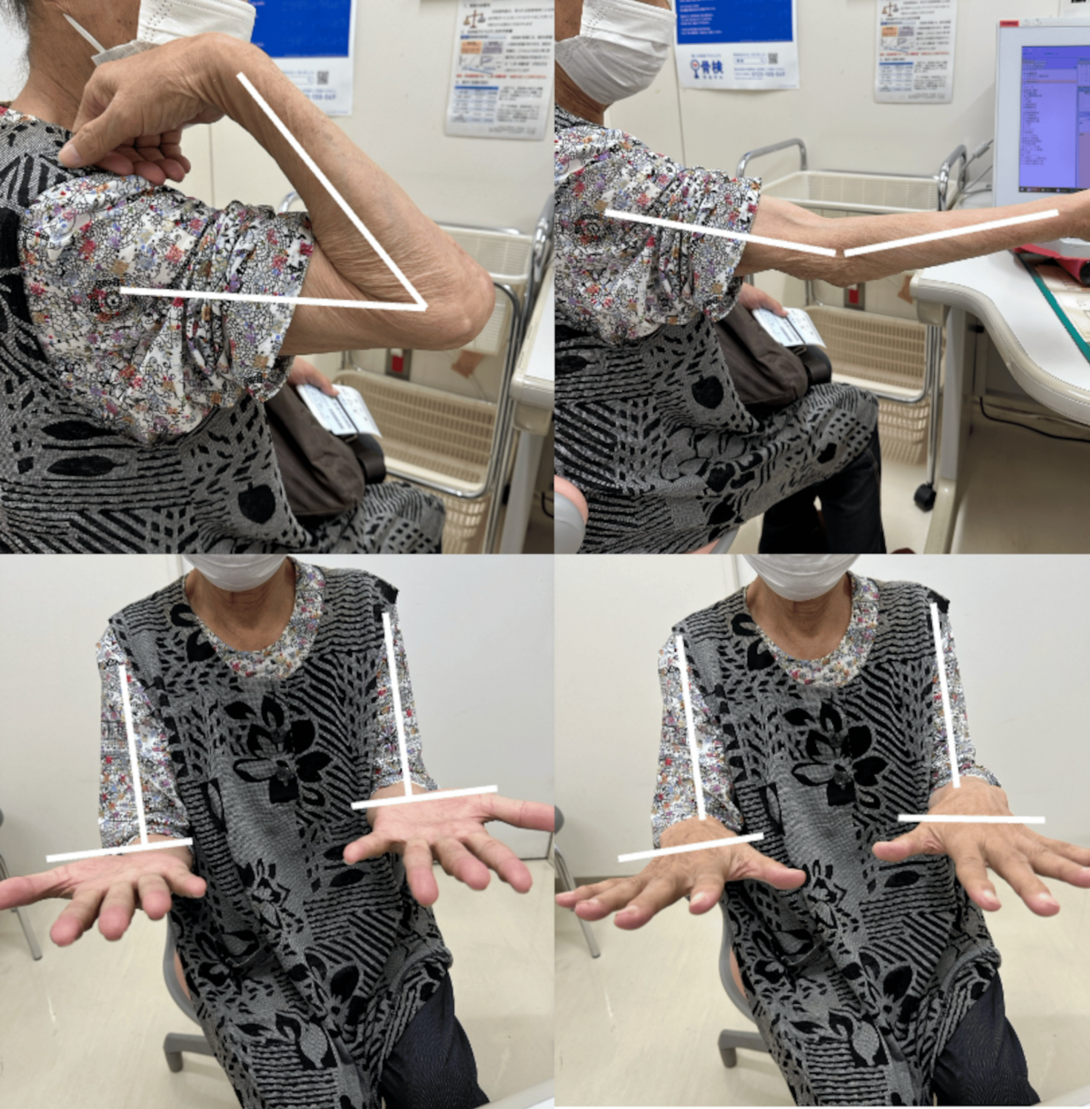
Range of Motion and Appearance at Final Follow-Up The range of motion included a 20-degree extension lag, 130 degrees of flexion, 90 degrees of supination, and 80 degrees of pronation. There was no varus-valgus instability of the elbow.

**Figure 6:**
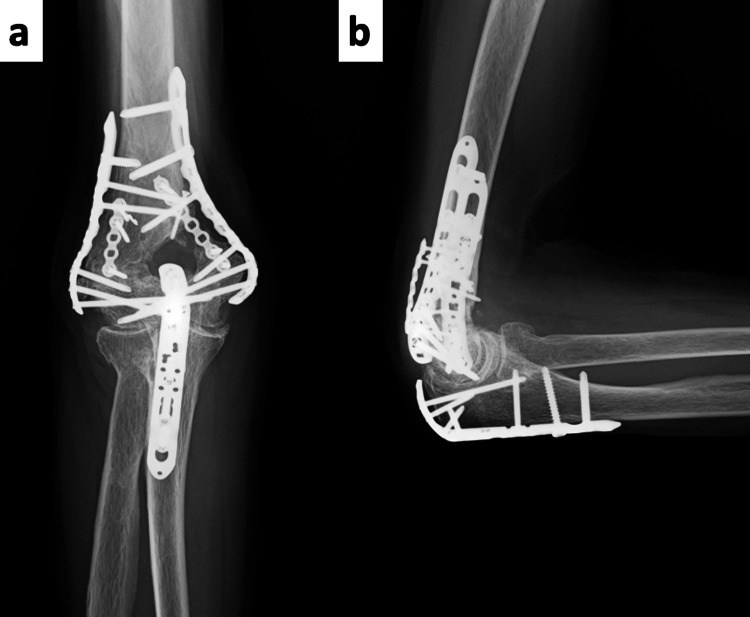
Final Follow-Up Plain Radiographs (a) Anteroposterior (AP) view; (b) lateral view. Bone union was confirmed, with no evidence of implant failure.

Postoperatively, the elbow was immobilized for two weeks, followed by an early range of motion exercises. Bone union was confirmed at four months postoperatively. At the final follow-up, she had no elbow pain at rest or during movement. The range of motion showed an extension lag of 20 degrees, 130 degrees flexion, 90 degrees supination, and 80 degrees pronation (Figures 5, 6). There was no varus-valgus instability of the elbow, and grip strength was 9.4 kg/9.0 kg. No limitations were observed in daily activities such as eating, dressing, or grooming. The Mayo Elbow Performance Score was 100 points, indicating an excellent outcome.

## Discussion

While there have been increasing reports of TEA for nonunion after distal humeral fractures in recent years [14,15], there are also reports that ORIF is possible using locking plates with simultaneous soft tissue release when bone morphology is preserved [18]. Some reports suggest that TEA is indicated for nonunion cases when bone morphology is not preserved [11,12].

In our case, although the articular surface was preserved (namely, the absence of osteoarthritis), there was a significant bone defect at the nonunion site, making both ORIF and TEA potential treatment options.

The patient specifically complained of symptoms when performing a push-off motion to stand up and during agricultural work. Considering that TEA typically has a weight-bearing limitation of 1-5 kgs [16], we determined that ORIF would likely provide better long-term outcomes if bone union could be achieved.

Cha et al. reported a method of excising sclerotic bone surfaces, correcting the alignment of the fracture site, and then fixing with locking plates [19]. According to Helfet's method for distal fragments, multiple drilling with 2-mm K-wires for decortication without extensive excision or opening of the proximal and distal ends, followed by block iliac bone grafting, has also been reported [9,10].

In our case, the bone ends were rounded, and complete resection would have resulted in a large bone defect. Therefore, similar to Helfet's approach, we performed decortication and transplanted block iliac bone, with additional mini-plate fixation to secure the grafted bone. Biomechanical studies have shown that parallel plating offers greater resistance to rotational deformation compared to 90-degree plating [20], so we also employed parallel plating.

Because bone union was achieved in this case, weight-bearing restrictions were unnecessary, allowing for a more confident follow-up, even in this elderly patient. Despite being 82 years old, she achieved bone union at four months postoperatively, and at the final follow-up, had no elbow pain at rest or during movement, with a good range of motion (extension lag of 20 degrees, flexion 140 degrees). The Mayo Elbow Performance Score was excellent at 100 points.

European studies comparing TEA and ORIF for distal humeral fractures have shown that despite no difference in functional outcomes, 63% of patients in the TEA group required reoperation, primarily due to periprosthetic fractures and implant loosening [11]. This supports the validity of choosing ORIF even for elderly patients with a life expectancy greater than five years, provided that bone quality is adequate.

As demonstrated in this case, appropriate bone fixation techniques can lead to good bone union and functional recovery, even in elderly patients. Additionally, a major advantage is that once bone union is achieved, there are no weight-bearing restrictions, allowing for a more natural daily life.

## Conclusions

We achieved good results with ORIF for distal humeral nonunion three years after injury. Appropriate treatment was possible by selecting the surgical technique after careful consideration of the patient's background. The keys to the success of ORIF include the absence of osteoarthritis changes in the articular surface and the absence of significant bone defects. Long-term follow-up is necessary in the future.
